# Walking Adaptability after a Stroke and Its Assessment in Clinical Settings

**DOI:** 10.1155/2014/591013

**Published:** 2014-08-28

**Authors:** Chitralakshmi K. Balasubramanian, David J. Clark, Emily J. Fox

**Affiliations:** ^1^Department of Clinical and Applied Movement Sciences, University of North Florida, 1 UNF Drive, Jacksonville, FL 32224, USA; ^2^Brain Rehabilitation Research Center (151A), Malcom Randall VA Medical Center, 1601 SW Archer Roadd, Gainesville, FL 32608, USA; ^3^Department of Aging and Geriatric Research, University of Florida, Gainesville, FL 32603, USA; ^4^Department of Physical Therapy, University of Florida, P.O. Box 100154, Gainesville, FL 32610-0154, USA; ^5^Brooks Rehabilitation, Jacksonville, FL 32216, USA

## Abstract

Control of walking has been described by a tripartite model consisting of stepping, equilibrium, and adaptability. This review focuses on walking adaptability, which is defined as the ability to modify walking to meet task goals and environmental demands. Walking adaptability is crucial to safe ambulation in the home and community environments and is often severely compromised after a stroke. Yet quantification of walking adaptability after stroke has received relatively little attention in the clinical setting. The objectives of this review were to examine the conceptual challenges for clinical measurement of walking adaptability and summarize the current state of clinical assessment for walking adaptability. We created nine domains of walking adaptability from dimensions of community mobility to address the conceptual challenges in measurement and reviewed performance-based clinical assessments of walking to determine if the assessments measure walking adaptability in these domains. Our literature review suggests the lack of a comprehensive well-tested clinical assessment tool for measuring walking adaptability. Accordingly, recommendations for the development of a comprehensive clinical assessment of walking adaptability after stroke have been presented. Such a clinical assessment will be essential for gauging recovery of walking adaptability with rehabilitation and for motivating novel strategies to enhance recovery of walking adaptability after stroke.

## 1. Introduction

Approximately, 600.000 individuals incur a stroke each year and stroke is the leading cause of long term disability in the United States [1, 2]. Walking function in those who have sustained a stroke may range from complete dependence to independent walking ability. During the first week after a stroke, only a third of persons are able to walk unaided [3] but at 3 weeks or at hospital discharge 50–80% of survivors can walk unaided [4, 5] and by 6 months approximately 85% of stroke survivors are able to walk independently without physical assistance from another person [6]. Interestingly, while up to 85% of individuals with a stroke regain independent walking ability [6–8], only about 7% of persons discharged from inpatient rehabilitation could manage steps and inclines and walk the speeds and distances required to walk competently in the community [8–10].

Walking in everyday life necessitates walking adaptability, which is the ability to modify walking to meet behavioral task goals and demands of the environment [11–13]. The ability to adapt walking is one component of a tripartite model of locomotor control, along with the ability to generate stepping and maintain postural equilibrium [11, 14]. Individuals with limited ability to appropriately adjust to changes in the task and environment may either choose to avoid walking in these contexts (a safety strategy) or experience a heightened risk of falls when required to walk under these challenging circumstances [15]. Indeed, the rates of falling are reported to be high, ranging between 23–34%, 40–73%, and 43–70% during a 3-4 month [7, 16], 6-month [17, 18], and 1-year follow-up [19, 20], respectively. Most falls are reported to result from a trip, a slip, or a misplaced step while walking [17, 21–24] and walking is also the most frequently reported activity (39%–90%) at the time of a fall in stroke survivors [7, 17, 25, 26], suggesting the reduced ability of individuals with stroke to adjust walking to task and environmental demands.

Despite the relevance of walking adaptability to everyday mobility and the reduced ability of individuals with stroke to adjust walking to task and environmental demands, assessment of walking adaptability has received relatively little attention. Frequently used assessments of walking recovery after stroke involve walking short distances (such as the 10 m walking speed test, timed up, and go test) and examination of isolated limb movements (e.g., Fugl-Meyer Assessment) to predict walking recovery [27–29]. Although valuable, these assessments do not account for the full repertoire of walking skills that are required to reengage in safe and independent ambulation in the home and community [29, 30]. Specific, comprehensive, and rigorous assessments for walking adaptability are essential to design targeted interventions to improve walking adaptability after stroke. Therefore, the purpose of this evidence-based review is to examine the challenges to clinical measurement of walking adaptability and to discuss the status of clinical measurement of walking adaptability after a stroke. Specifically, we aimed to identify existing performance-based clinical assessments of walking function that can measure adaptability and in turn inform rehabilitation strategies to improve recovery of walking adaptability after stroke. This review is organized in five sections. First, we present the neural control model of walking which identifies adaptability as a distinct requirement for optimal walking function. Second, we discuss the conceptual challenges in measurement of walking adaptability and propose some solutions to these challenges. Third, we review the existing literature related to experimental evidence quantifying limitations and capacity in walking adaptability after stroke. Fourth, we review the content of current performance-based clinical assessments of walking function to determine the extent to which they capture the construct of adaptability. Finally, we have proposed some strategies for effectively measuring walking adaptability in clinical settings for individuals with a stroke.

## 2. Neural Control Model of Walking

**Figure 1:**
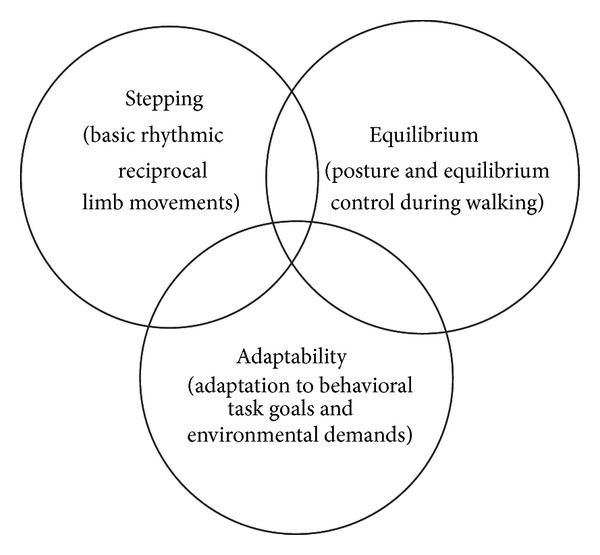
Neural control model of functional walking. Neural control of walking can be explained as a tripartite model consisting of stepping, equilibrium, and adaptability [11, 14]. All three are necessary for optimal walking function. This review focuses on walking adaptability, which is defined as the ability to adjust walking to behavioral task goals and environmental circumstances.

A major rationale for the need to measure walking adaptability separately from steady-state walking is that adaptability has some distinct neural control requirements. Among the earliest works identifying adaptability as an independent neural control construct is the framework proposed by Forssberg [11] and Grillner and Wallen [14]. This framework identifies the three primary requirements for the CNS to generate purposeful or goal-directed locomotion: stepping, equilibrium, and adaptability (Figure 1). First, the CNS must generate the basic stepping pattern of rhythmic reciprocal limb movements while supporting the body against gravity and propelling it forward. Second, the CNS must maintain control of equilibrium to keep the center of mass over a constantly moving base of support and maintain the body upright in space. Finally, the CNS must have some adaptive capabilities for locomotor control so that the basic pattern can be adapted according to the environmental circumstance or changes in the behavioral goal. Walking adaptability conceptualizes the third essential requirement for successful walking as earlier proposed (Figure 1).

Accumulating evidence further supports a model of walking that distinguishes some of the neural control requirements of walking adaptability from stepping and balance. The basic reciprocal pattern of stepping is primarily controlled by pattern generating circuits in the spinal cord [33, 34]. For typical steady state walking, these circuits are activated by an indirect locomotor pathway believed to include the motor cortex, basal ganglia, and brainstem locomotor centers [35, 36], as well as by signals from the cerebellum and the periphery (e.g., sensorimotor reflexes) [37]. While typical steady state walking involves some direct control from the corticospinal pathway [38–41], the role of corticospinal control is known to be much greater for tasks requiring adaptability [42–47]. For example, evidence from animals shows that central motor lesions severely limit the capacity for adaptability [44], while stepping is relatively preserved [48, 49]. Additional evidence from cat studies shows that corticospinal neurons increase their firing during ladder walking, obstacle crossing, and obstacle avoidance [50–52]. Analogous results have been shown in humans, as motor evoked potentials are increased in leg muscles during walking tasks requiring accurate control [53, 54]. Based on this cumulative evidence, stroke would be expected to be particularly detrimental to walking adaptability because a stroke often directly damages the supraspinal motor pathways (i.e., motor cortex, cerebral white matter, and/or internal capsule) that are critical for walking adaptability. Since walking adaptability is also critical for the optimal functional recovery of walking function, measurement of walking adaptability after stroke warrants attention.

## 3. Conceptual Challenges for the Measurement of Walking Adaptability

Measurement of walking adaptability has received relatively little attention in clinical settings, as indicated in part by the absence of a comprehensive, well-accepted assessment of walking adaptability. A number of factors may account for the lack of progress in this area. First, there is no uniform terminology to define the construct of walking adaptability. Second, the construct of walking adaptability covers a broad spectrum of situations because adaptations of walking may be required under varied task and environmental demands. Clinical assessments of walking adaptability must therefore reflect this multi-faceted and complex nature of adaptability.

### 3.1. Lack of Uniform Terminology

While walking adaptability has been the subject of interest in several studies, there is a lack of uniform terminology used to describe the construct. The most common task paradigms that refer to similar constructs as walking adaptability are “gait adaptability,” “obstacle crossing/clearance/negotiation,” and “locomotor adaptation.” Houdijk and colleagues define “gait adaptability” as “the ability to adjust gait to environmental circumstances, such as obstacles and targets” [55]. They have studied gait adaptability as the adjustments in foot placement in response to visual or acoustic stimuli delivered during treadmill walking. However, walking adaptability may not necessarily require overt changes to the walking pattern. Rather, it may require motor control adjustments that (in some cases) preserve the mechanics of walking such as when walking under different ambient conditions or constraints posed by the terrain. “Obstacle crossing/negotiation/clearance” is another term used in the literature to indicate walking adaptability [23, 56–64]. This term has been most frequently utilized in the body of work investigating the mechanisms related to obstacle clearance. Avoiding obstacles is an important and unique dimension of walking adaptability, but “obstacle avoidance” does not comprehensively capture the multi-faceted construct of walking adaptability. “Locomotor adaptation,” another term selectively used in the work by Bastian and colleagues, refers to an error driven motor learning process that is used to alter the spatiotemporal elements of walking [65, 66]. Bastian and colleagues utilize a split-belt treadmill to study locomotor adaptation and their conceptualization of locomotor adaptation encompasses an important aspect related to learning of walking adjustments over time [66, 67].

Our conceptualization of walking adaptability is founded on the previously mentioned tripartite locomotor control model of stepping, equilibrium, and adaptability. Terminology based on this fundamental neural control model provides a strong foundation and a broader context for the use of the term, “walking adaptability.” Furthermore, since emerging rehabilitation paradigms have utilized and focused on this tripartite model [12, 13, 68], our approach is intended to synchronize with these paradigms in order to provide a uniform framework for both assessment and intervention of walking adaptability after stroke.

### 3.2. Complex Nature of the Construct of Walking Adaptability

**Figure 2:**
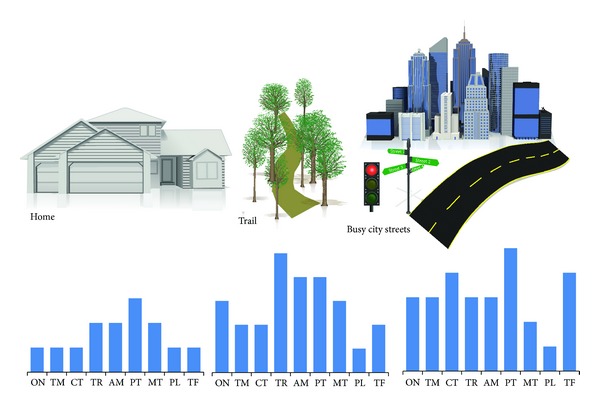
Conceptual illustration of the domains of adaptability. This figure illustrates the relative demands that may be placed on the nine domains of walking adaptability in different ambulatory environments. The nine domains of walking adaptability have been adapted from earlier work by Patla and Shumway-Cook [15]. In a less complex and predictable environment such as the home, the requirements for walking adaptability would be less demanding and encompass fewer domains relative to more challenging environments such as walking on a nature trail or on a busy city street. Abbreviations: ON—obstacle negotiation; TM—temporal demands; CT—cognitive dual-tasking; TR—terrain demands; AM—ambient demands; PT—postural transitions demands; MT—motor dual-tasking; PL—physical load; TF—maneuvering traffic.

Walking adaptability is necessitated when the complexity of the task situation exceeds what can be accomplished with basic stepping. For example, walking adaptability is crucial when walking on uneven or cluttered terrains (unpredictable environment), to ensure safe and appropriate foot placement [64, 69]. Similarly, walking adaptability is essential when the task requires walking and turning to negotiate a curved path. There can be numerous combinations of task goals and environmental circumstances that need to be considered to comprehensively capture walking adaptability (Figure 2). A starting point to capture the different situations and contexts necessitating walking adaptability is the conceptual framework developed by Patla and Shumway-Cook [15]. Patla and Shumway-Cook describe eight dimensions that impact a person's ability to interact in the environment and their framework includes time constraints, distance, ambient conditions, terrain characteristics, external physical load, attentional demand, postural transitions, and traffic density [15, 70]. Although this framework was originally intended to characterize the external demands of community ambulation, it also can be applied to characterize the spectrum of environmental situations that require walking adaptability. Thus, our application of this framework to the topic of “walking adaptability” is inclusive of all ambulation settings, not just community ambulation. Based on Patla and Shumway-Cook's framework [15], we propose nine domains of walking adaptability that are defined in Table 1.

While we primarily conceptualized the domains of adaptability based on Patla and Shumway-Cook's proposed dimensions of community mobility, there are some differences in our approach. First, we conceptualize the “domains” of walking adaptability as the “capacity/ability” of an individual (an internal characteristic) as opposed to Patla and Shumway-Cook's emphasis of the environmental context (an external characteristic). Second, we incorporated seven of the eight dimensions of community mobility proposed by Patla and Shumway-Cook, excluded one dimension, and modified two dimensions to create overall nine domains of walking adaptability. Particularly, we modified the “traffic density” dimension of Patla and Shumway-Cook's framework [15] and subcategorized this dimension into two domains of  “obstacle negotiation” and “maneuvering in traffic.” Amassing literature (discussed below) suggests unique limitations of individuals with stroke when negotiating obstacles warranting an exclusive assessment of this domain of walking adaptability. “Maneuvering in traffic” refers to successfully avoiding collision with obstacles by maneuvering the entire body and may require discrete set of abilities (like processing the speed and direction of movement) when compared to stepping over an obstacle. Therefore, we propose that “maneuvering in traffic” should be assessed as a separate domain of walking adaptability. We also modified the “attentional demands” dimension of Patla and Shumway-Cook's framework [15] and subcategorized this dimension into two domains of “cognitive dual-tasking” and “motor dual-tasking.” Since the addition of a cognitive or motor task to walking may demand unique and varying amounts of attention resources, we propose that adjusting walking to cognitive and motor dual-tasks necessitates distinct measurement. We also excluded the dimension of “distance” originally proposed by Patla and Shumway-Cook since distance pertains to endurance of walking and does not necessitate adaptations.

Each of these domains represents a demand (goal of the task or an environmental constraint) that may necessitate walking adaptability. Figure 2 is a conceptual illustration of the domains of walking adaptability in varying environments. In any given environment, the demand on a particular domain and the number of domains included may vary (Figure 2). For instance, in a less complex and predictable environment, fewer domains may be represented and those that are represented may have lower demand. An example is walking at home with uncluttered walking paths, walking at one's own pace and with no traffic encountered. In contrast, a complex and unpredictable environment such as a busy city street will involve greater representation of more number of domains for negotiating street curbs, ramps, walking to cross a street, conversing with a friend while walking, walking at dusk or when raining, holding to shopping bags while walking, and maneuvering around other people (Figure 2). An advantage of using these domains to define task goals and environmental demands that necessitate walking adaptability is that it is not specific to the environment in which walking is being performed but, instead, is reflective of the demands for walking adaptability under varied situations (Figure 2).

## 4. Evidence of Impairments in Walking Adaptability in Individuals with Stroke

The domains of walking adaptability most notably investigated and reported in the stroke literature include “obstacle negotiation,” “temporal demands,” and “cognitive dual-task demands.” There is also emerging evidence pertaining to “terrain demands” and “ambient demands.” Other domains have received some attention as components of clinical assessments, but specific performance results for persons after stroke are not readily available in the literature. These include the domains of “postural transitions,” “motor dual-task demands,” “physical load,” and “maneuvering in traffic.” Here, we review some of the most pertinent evidence highlighting the impairments in walking adaptability after stroke.

### 4.1. Obstacle Negotiation

The capability to negotiate obstacles in the environment is crucial for safety during walking. Obstacle negotiation involves modifying the typical gait pattern to prevent a collision between the lower limb and the obstacle. This often requires a well-timed coordination between the visual and motor systems in order to produce an appropriate limb trajectory while maintaining dynamic balance. Limitations in walking adaptability using obstacle clearance paradigms have been well studied in the stroke population. Individuals with a stroke have greater failures rates when avoiding obstacles despite using a more cautious strategy of stepping over with a higher toe clearance with the lead limb [56]. Additionally, Said and colleagues reported that although the lead limb clearance was high, the limb trajectory was much more variable increasing the stability demands of the trail limb [99]. While the failure rate was lower when the unaffected limb crossed the obstacle first, individuals with stroke showed no preference in crossing with the affected or unaffected limb [56, 58]. Toe clearance of the trail limb was also shown to be reduced, increasing the risk of tripping [23]. Individuals with stroke also demonstrate increased anterior-posterior separation of the COP and COM when negotiating obstacles, suggesting greater instability during the task [57]. Furthermore, individuals with stroke show inaccurate foot placement of the affected lead foot when clearing obstacles and prefer using a step lengthening strategy [58, 59].

The deficits in adjusting foot placement when clearing obstacles are shown to be even more prominent when executed under time pressure as when individuals had to avoid obstacles that suddenly appeared before them [58, 100, 101]. Furthermore, adding a cognitive task during obstacle crossing deteriorated performance in individuals with stroke and increased their risk of obstacle-heel contact during crossing [61]. Individuals with stroke also seemed to utilize a “posture-first strategy” even when they were instructed to keep up their performance on both motor and cognitive tasks [59]. Moreover, van Swigchem and colleagues recently identified motor impairments associated with the difficulty in obstacle avoidance after stroke [64]. They reported that individuals with stroke demonstrated a delay and reduction in muscle responses and therefore suggested that rehabilitation interventions that aim to improve obstacle avoidance should focus on time-critical obstacle training.

### 4.2. Temporal Demands

Walking tasks often require time constraints, such as needing to walk faster when crossing a busy intersection or when walking to answer a ringing phone. In some cases, temporal demands might also require slower walking such as when walking with a slow moving crowd. A number of studies demonstrate the compromised ability of individuals after stroke to increase walking speed over and above their self-selected speed [102–104], which is expected given that self-selected speeds are well below normal. While most rehabilitation studies have focused on improving temporal walking performance (i.e., gait speed) as the primary outcome measure and walking speed has also shown to increase with rehabilitation [102, 105, 106], walking speeds generally remain well below normal and do not transfer to substantial gains in home and community ambulation [9, 10]. These findings argue for the need to broaden rehabilitation interventions and assessments to account for other aspects of walking adaptability that may contribute to functional gains.

### 4.3. Cognitive Dual-Tasking

Secondary task demands involve combining walking with other attention-demanding tasks such as engaging in conversation, reading a map, and so forth. This is commonly referred to as “dual-tasking,” and is known to degrade walking performance even in healthy adults [107]. After a stroke, dual-tasking conditions of walking and concurrently performing a cognitive task have shown to profoundly impact the walking performance compared to age-matched healthy controls [108–111]. Walking and concurrently performing a cognitive dual-task reduces gait speed [108–110], stride duration [108, 111, 112], stride length [108, 109], double support time [110, 113], and cadence [108, 114]. The results of cognitive-motor interference in individuals with stroke are consistent across the use of different secondary cognitive tasks involving a range of cognitive functions (such as working memory, executive function, visuospatial processing, and language) [107, 115]. Moreover, the majority of the research demonstrating such cognitive-motor interference reports that individuals with stroke prioritize the cognitive task, sacrificing walking performance [108, 109, 111, 112], suggesting the impaired ability to adapt the walking pattern in the presence of a cognitive task.

### 4.4. Terrain Demands

Terrain refers to walking on surfaces that are not flat and firm. This includes stairs, escalators, ramps, curbs, side slopes, grass, sand, trails with tree roots, and so forth. Relatively little work has been conducted to assess the capability of individuals after stroke to adapt walking to challenging terrain conditions. Phan and colleagues [116] showed that individuals with stroke reduced their walking speed and step length when walking downhill compared to both level and uphill walking. Walking speed in healthy controls, however, remained unchanged across different conditions. A number of studies have documented deficits in stair ascent/descent performance after stroke [117, 118] and at least one study has developed a clinical assessment of stair performance [119].

### 4.5. Ambient Demands

Ambient conditions refer to factors such as lighting, temperature, weather conditions, noise levels, familiarity with surroundings, and other such factors that may interact with walking. Although most of these factors have not been specifically examined, there is emerging evidence that the ambient environment, in general, can influence walking function. One recent study found that faster walking speeds were obtained when stroke participants walked outdoors relative to walking indoors [120]. Other studies have also reported that walking speed was lower when tested in a shopping mall compared to on a street [121] or in a clinic [122], suggesting that the ambient environment can influence walking function. Clinic-based walking speed assessment predicts community walking speeds for faster walking stroke survivors, but not for slower walking stroke survivors [123].

### 4.6. Less Investigated Domains of Walking Adaptability

There is a need for research to examine poststroke deficits in the domains of “postural transitions,” “motor dual-tasking,” “physical load,” and “maneuvering in traffic.” Postural transitioning refers to the capability of an individual to alter body orientation or head position in order to perform a task, such as turning a corner, observing the surrounding environment (i.e., head turns), reaching during walking, bending during walking, or walking through a narrow space. Motor dual-task includes such tasks as carrying a tray of food and dialing a phone while walking. Like cognitive dual-task, this involves a competition for attentional resources. However, manual dual-tasking may also involve a competition for resources (such as motor programs) that are detrimental to safe control of walking. Physical load demands while walking include tasks such as opening a heavy door, walking while wearing a heavy backpack, or carrying a weighted package. Interaction with a physical load during walking necessitates successfully adapting to any changes to the walking pattern (e.g., perturbations to the body mass) created by the additional physical load. Maneuvering in traffic can include factors such as other people (i.e., when walking on a busy sidewalk), large environmental objects (i.e., street signs, furniture), or pets in the home. In some cases, maneuvering may require some online processing of information about speed and direction of movement, in order to predict where the object is likely to move relative to oneself. Since maneuvering in traffic may require discrete set of abilities (like processing the speed and direction of movement), we propose its assessment as a separate domain of walking adaptability.

It is important to consider that walking adaptability tasks in each of the proposed domains require the integration of all three neural control requirements of walking: stepping, equilibrium, and adaptability. While the limitations in steady-state walking ability after a stroke due to compromised stepping and equilibrium are well characterized [5], quantification of limitations in walking adaptability is not well established and warrants further work. To date, the existing limited experimental evidence highlights the limitations in adaptability in individuals with stroke, thereby supporting the need for individualized assessment of these limitations.

## 5. Existing Performance-Based Clinical Assessments of Walking Function and the Measurement of Walking Adaptability Post-Stroke

Since there is no gold-standard clinical assessment of walking adaptability, performance-based clinical assessments of walking function were reviewed to determine the extent to which they measure the construct of walking adaptability. Assessments included in this review contained at least a subset of walking activities requiring adaptability, had at least one peer-reviewed study with detailed information about the content of the assessment, were developed for any adult population (not limited to stroke), and were clinically feasible. While some of the clinical assessments included in our review may lack recommendation for clinical use based on earlier published criteria [124], our purpose in reporting these was to provide an in-depth review of the content of these tools and identify the domains of walking adaptability that they may capture. We excluded four assessments (balance evaluation systems test, timed up and go measure, Fullerton advanced balance scale, and stops walking when talking) from our review since all items contained in these assessments were redundant with clinical assessments included in this review.

### 5.1. Dynamic Gait Index

The dynamic gait index (DGI) was developed to assess an individual's ability to modify gait in response to changes in task demands [74]. The DGI was originally developed for community-dwelling older adults and consists of 8 walking tasks requiring persons to modify their gait to varying demands, such as walking at different speeds, walking while turning head, ambulating over and around obstacles, stair negotiation, and making quick turns. The performance on the items of the DGI is measured on a 4-point ordinal scale (0–4), with higher scores indicating better performance and a maximum score of 24 [74]. The psychometric properties of the DGI have been well tested with several reports of reliability, validity, and sensitivity in varied clinical populations, including stroke [71–73, 75–79, 125], as presented in Table 2. Seven of the eight items on the DGI assessment involve walking adaptability capturing 5 domains of walking adaptability (obstacle negotiation, postural transitions, temporal demands, terrain demands, and maneuvering in traffic) (Table 3).

### 5.2. Functional Gait Assessment

The functional gait assessment (FGA) is based on the DGI and was developed for persons with vestibular disorders to reduce the ceiling effect of the DGI for this population [85]. The FGA is a 10-item walking test comprising of 7 of the 8 items from the original DGI and 3 new items, including walking with a narrow support base, walking backwards, and walking with eyes closed. Similar to the DGI, the performance on the items of the FGA is measured on a 4-point ordinal scale (0–4), with a maximum score of 30 [85]. The FGA like the DGI has also been tested in several clinical populations, including the stroke population [71, 79–84]. Additionally, psychometric properties of reliability, validity, and sensitivity have been tested (Table 2). The FGA also reduces the ceiling effect of the DGI in persons with stroke [71]. The items of the FGA capture four domains of walking adaptability, to include obstacle negotiation, postural transitions, temporal demands, and terrain demands. Two of the three new items of the FGA add measurement in the domain of postural transitions (Table 3).

### 5.3. Modified Emory Functional Ambulation Profile

The modified Emory functional ambulation profile (mEFAP) is a modification of the functional ambulation profile, is a timed measure of walking under five environmental challenges, and was developed for individuals with stroke [87]. The mEFAP challenges a person to walk over a standardized array of terrains (floor, carpet, and stairs), step over obstacles and walk with postural transitions (rise from a chair and walk a distance). Each of the items on the mEFAP is timed and scored based on standardized criterion. Individuals' time to complete each subtask is recorded and this time is multiplied by a factor assigned based on the use and type of an assistive device [87]. The total score is the summed total for each subtask. The mEFAP has been exclusively tested in the stroke population and reliability and validity for use in individuals with stroke has been reported [86, 87], as shown in Table 2. Four of the five items of the mEFAP measure walking adaptability but only a limited number of domains of walking adaptability (terrain, obstacle negotiation, postural transitions, and maneuvering in traffic) are captured (Table 3).

### 5.4. Spinal-Cord Injury Functional Ambulation Profile

The spinal-cord injury functional ambulation profile (SCI-FAP) is modified from the mEFAP and is a timed measure of functional walking developed for individuals with an incomplete spinal cord injury [89]. The SCI-FAP is a 7-item assessment scale that comprises of 4 items similar to the mEFAP and 3 new items (walking to step up on a small step and continue walking, walking while carrying a shoulder bag and walking to open a door and continue walking through). Scoring of the SCI-FAP is similar to that of the mEFAP but the maximum possible score is higher due to the addition of the 3 items. While reliability and sensitivity of the SCI-FAP have been reported in individuals with incomplete SCI [88, 89], as shown in Table 2, validity of the SCI-FAP has not yet been established. While this assessment tool was originally developed for individuals with SCIs, some of the additional items on the SCI-FAP capture additional domains of walking adaptability. Specifically, the SCI-FAP captures the domain of adapting walking to interact with a physical load, one of the domains that has been less frequently assessed. The SCI-FAP adds measurement of one more domain of walking adaptability (physical load) when compared to the mEFAP (Table 3).

### 5.5. High-Level Mobility Assessment Test

The high-level mobility assessment test (Hi-MAT) was developed for persons with traumatic brain injury (TBI) to assess high-level mobility skills [90, 91]. The Hi-MAT consists of 13 items that assess balance and mobility utilizing a wide-range of high-level activities such as walking, stair negotiation, running, hopping, skipping, and jumping. Majority of items (excluding bounding and stair items) are performed by the individuals at their fastest safe speed and the performance times and distances (raw scores) are recorded. The raw scores are converted to a score from 0 to 4 using a standardized scoring table and the item scores are summed to produce a total score, with a maximum score of 54. The Hi-MAT has primarily been tested in persons with acquired and traumatic brain injuries and reliability, validity, and sensitivity have been reported for this population (Table 2). Normative data of performance have also been reported [126]. The Hi-MAT has not yet been tested in the stroke population. While the walking items on the Hi-MAT are similar to other clinical assessments, these activities (except stair negotiation) are tested under time constraints (maximal safe speed) and may provide a sensitive method to unmask adaptability deficits in some higher-functioning individuals. Recent evidence shows that adaptive responses are not only impaired but also delayed in individuals with stroke, suggesting that walking adaptability should be assessed under time constraints to sensitively identify limitations [64]. Seven of the 13 items on the Hi-MAT measure walking adaptability but only 4 domains of walking adaptability (temporal demands, terrain, and postural transitions) are captured by these items (Table 3).

### 5.6. Community, Balance, and Mobility Scale

Similar to the Hi-MAT, the community balance and mobility (CB&M) scale is a relatively challenging assessment and is reported to evaluate high-level deficits in gait, balance, and mobility [95]. The CB&M scale was originally developed for and validated in high-functioning young and middle-aged ambulatory adults with TBI but validity of the scale has also been investigated in the stroke population [96]. Reliability and validity have also been reported for community-dwelling older adults (ref). The CB&M scale incorporates several demanding walking tasks commonly performed in the community environment like walking and looking at a visual target with and without carrying weighted bags, walking and bending to pick up an object, rising and continue walking. Walking forward turning and walking backwards, tandem walking, and stair negotiation with and without a secondary manual task. Other challenging activities included in the CB&M scale measure balance (e.g., holding balance on one leg, pivoting and scooting on one leg, hopping, running, and standing step-ups). The items are scored on a 0–5-ordinal scale (except one item that is scored on a 0–6-ordinal scale), with an extra point given for carrying a basket while descending stairs. The maximum score on the CB&M scale is 96. The CB&M scale has been tested in several clinical populations, including one report in the stroke population [92–96], as presented in Table 2. Six of the 13 items on the CB&M measure walking adaptability capturing 4 domains (postural transitions, temporal demands, terrain, and motor task) (Table 3). The CB&M scale includes some tasks requiring adaptability in more than one domain, which could enhance its relevance for capturing the complexity of walking adaptability.

### 5.7. Sensory-Oriented Mobility Assessment Instrument

The sensory-oriented mobility assessment instrument (SOMAI) was designed to understand how sensory inputs are utilized in mobility performance and included modifications of assessments to create the tasks for this test [98]. The SOMAI consists of 10 items, referred to as “maneuvers”. The manuevers are carried out sequentially with individuals rising from a chair, walking down an 8.25 m path, reaching up, bending down, turning 180 degrees, walking on a carpet surface and negotiating 2 foam surfaces placed sequentially. The maneuvers are performed twice, first with normal vision and next wearing goggles (focal vision) to eliminate peripheral vision. Test performance is graded using a 0–3 ordinal scale, with higher scores representing greater impairment. While specific reports on psychometric properties (such as reliability, sensitivity, and so forth) of this assessment do not exist, authors who developed the assessment validated the assessment as a tool for examining the effect of sensory systems on functional mobility [98], (Table 2). We included the SOMAI in this review since it requires rapid adaptations to changing environmental and task demands like walking to step up on foam under normal and reduced vision (aspects of adaptability not captured by other assessments). Eight of the 20 items measure walking adaptability, but only 2 domains of terrain and ambient demands are captured by these items.

### 5.8. Walking InCHIANTI Toolkit

The walking InCHIANTI toolkit (WIT) is a battery of 14 standardized walking tests developed to capture the typical conditions encountered in daily life. The battery was developed in association with the InCHIANTI study, an epidemiologic study of risk factors for mobility disability in old age [97]. The walking tests include simple walking tasks such as usual and fast-pace walking and more complex walking tasks that require adaptations including narrowing support base while walking at a fast pace, taking long steps while walking, walking fast to step over obstacles, walking and carrying a large but light package, walking while simultaneously performing a secondary cognitive task, walking and picking up an object from the floor, walking a long distance (400 m) fast, and walking fast while wearing a weighted-jacket. The battery of walking tests is based on a person-environment interaction model of mobility disability, developed by Patla and Shumway-Cook [15]. The 14 walking tests are performed over a specified distance, from 4 to 400 m, and each item is timed. Normative values based on the results of the InCHIANTI study population of ~1200 men and women, and ages 20–85 are available [127]. Reliability of the WIT test is reported for community-dwelling older adults but no other psychometric properties have been tested (Table 2). The WIT includes several complex tasks that capture multiple domains of adaptability simultaneously. Twelve of the 14 tasks included in the WIT necessitate adaptations while walking and capture 7 domains of adaptability (obstacle negotiation, temporal demands, ambient demands, postural transitions, motor dual-tasking, and physical load) (Table 3).

### 5.9. Obstacle Course Tests

Two obstacle course-based assessments have been developed and validated in elderly, community-dwelling, and primarily male study populations (mean age = 75 years) [31, 32]. Rubenstein and colleagues developed a standardized walking obstacle course (SWOC) designed to simulate activities representing the mobility requirements in real-life situations [31]. The 31 m obstacle course includes 6 different tasks to test balance, gait, and functional mobility and performance of the course is timed. The tasks include tandem walking, walking on foam, ramps, and stepping over obstacles. Reliability and validity of the SWOC have been reported in community-dwelling older adult men but the SWOC has not been tested in other clinical populations (Table 2). The developers of the assessment designed the SWOC to mimic real-life situations and the SWOC consists of several walking adaptability tasks. All of the six items of the SWOC measure walking adaptability, but these items capture only 3 domains of walking adaptability (terrain, postural transitions, and motor dual-task) (Table 3).

The obstacle course developed and validated by Means and colleagues consists of a series of 12 stations where functional tasks commonly encountered in the home environment are presented [32]. The stations were designed to challenge different balance and walking strategies and include 4 stations with different floor surfaces, 2 ramps and 2 sets of stairs, and 4 functional tasks which include rising from a chair, opening a door, and stepping over obstacles. Performance on the obstacle course is assessed by measuring the time to complete the course, and the video-recorded performance on each station is scored on an ordinal scale (0–3) based on whether the task could be completed (unable to complete without assistance = 0) or if the task was performed with difficulty (no observed difficulty = 3) (Means KM 1996). The obstacle course has been validated in community-dwelling elderly. It has not been tested in other clinical populations and currently lacks testing of psychometric properties like reliability and sensitivity (Table 2). All of the 12 items of the obstacle course test measure walking adaptability, capturing the 5 domains of walking adaptability (terrain, obstacle negotiation, postural transitions, motor dual-tasking, and maneuvering in traffic) (Table 3).

### 5.10. Multiple Task Test

The multiple tasks test (MTT) is a test that simultaneously assesses the multiple components of postural control and was developed for individuals with Parkinson's disease [128]. The MTT consists of 8 sequential tasks of increasing difficulty with get up and go serving as the baseline which was repeated in all the tests. The baseline get up and go task involved getting up from a chair, walking a predefined course, turning 180 degrees, and coming back to sit in a chair. The tasks in the MTT include get up and go and then seven tasks with get up and go, answering a continuous series of questions, avoiding obstacles, carrying an empty tray, carrying a loaded tray, wearing indoor shoes with slippery soles, tip the floor halfway (i.e., squatting and touching the floor), and wearing sunglasses dimming the illumination. Tasks are scored both qualitatively and quantitatively. Qualitative scoring involved grading errors defined as normal, hesitations (slowed performance), or blocks (complete cessation) [128]. Persons with PD make more errors than young and elderly controls and errors increased as the tasks became more complex, suggesting it as an assessment of postural control and balance in persons with PD [129]. However, there are not yet reports on specific psychometric properties of the MTT in PD or any other pathologic diagnoses. The MTT includes items assessing multiple domains simultaneously and sequentially increases the difficulty level of assessment. All of the 8 items on the MTT measure walking adaptability, capturing the 7 domains of walking adaptability (postural transitions, obstacle negotiation, cognitive task, terrain demands, ambient demands, motor task, and maneuvering in traffic) (Table 3).

### 5.11. Summary from the Review of Existing Performance-Based Walking Assessments

**Figure 3:**
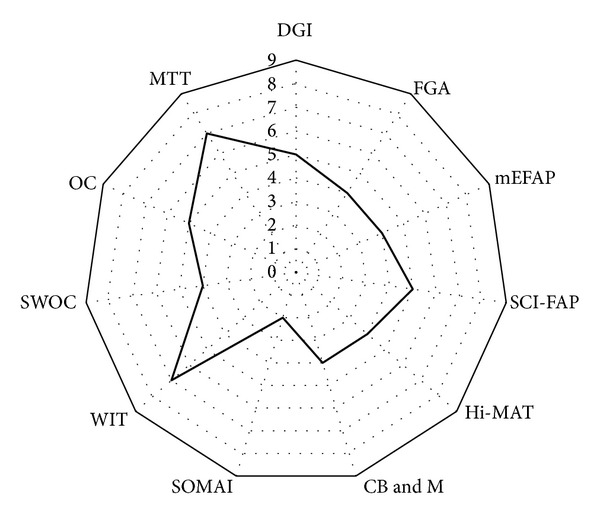
Number of walking adaptability domains captured by clinical assessments of walking function. The number of domains captured by clinical assessments range from 2 to 7, with the SOMAI capturing the least number of domains and the MTT and WTT capturing the most number of domains. Abbreviations: DGI—dynamic gait index; FGA—functional gait assessment; mEFAP—modified Emory functional ambulation profile; SCI-FAP—spinal cord injury functional ambulation profile; Hi-MAT—high-level mobility assessment test; CB&M—community balance and mobility scale; SOMAI—sensory-oriented mobility assessment instrument; WIT—walking InCHIANTI toolkit; SWOC—standardized walking obstacle course developed by Rubenstein and colleagues [31]; Obstacle course—obstacle course developed by Means [32]; MTT—multiple task test.

First, the review of performance-based clinical assessments indicates that there are several existing performance-based clinical assessments that capture aspects of walking adaptability and a pool of assessment items (from existing clinical assessment tools of walking) may be available for the measurement of walking adaptability. However, there is lack of a comprehensive assessment of walking adaptability, with most assessments capturing only 4-5 domains of walking adaptability. The WIT and MTT measured the most number of domains of walking adaptability (7 out of 9 domains) compared to other assessments but there is very limited testing of these assessments and it is unknown if these tests are appropriate for clinical populations with diverse mobility limitations, including stroke (Figure 3, Table 2). Second, only four of the assessments (DGI, FGA, mEFAP, and CB&M) reviewed here have been tested in the stroke population, with the DGI having undergone the most extensive testing in the stroke population across different stages of stroke recovery (Table 2). Third, rigorous assessment of walking adaptability was lacking, as evidenced by measurement of successively and hierarchically increasing difficulty levels necessitating walking adaptability. Hierarchical administration of walking adaptability tasks can rigorously challenge the individual unmasking adaptability limitations in a patient-specific manner (i.e., an individual with greater ability level may not present any limitations unless a more challenging task is administered). The MTT assessment proposes an underlying hierarchy but it lacks sufficient behavioral outcomes to validate the hierarchy. Additionally, the instrument was originally developed for individuals with Parkinson's disease and it is unclear if the proposed hierarchy would be pertinent to walking adaptability and to individuals with stroke.

## 6. Recommendations for Clinical Measurement of Walking Adaptability

The lack of a comprehensive, rigorous, and well-accepted clinical assessment for measuring adaptability after stroke implies the fact that it is difficult to assess the extent to which different rehabilitation approaches are effective for inducing recovery of adaptability. A rigorous and comprehensive clinical assessment of walking adaptability will also provide clinicians with an objective measure to accurately assess adaptability performance and recovery, thereby motivating the design of novel interventions to enhance recovery of adaptability. Therefore, there is an urgent need for the development and validation of a comprehensive multidomain and rigorous clinical assessment of walking adaptability after stroke. Development of such a clinical assessment may not necessarily require designing entirely new assessment items since our review shows that currently a pool of assessment items are available in existing clinical walking assessments. However, it may be essential to develop a comprehensive and rigorous tool by validating relevant items for adaptability measurement after stroke and developing hierarchically ordered assessment items. A comprehensive assessment consisting of different domains of task and environmental demands necessitating adaptability can prompt the clinician to administer the most relevant domains and assess patient strategies and performance. Choice for the administration of specific domains could be based on factors like (a) the individual's unique task and environmental constraints and (b) patient's self-report of limitations in specific domains. For example, for an 85-year-old woman after stroke, living independently at home with hard wood flooring, selecting assessment items from the terrain domain may be most relevant. Combining items on this domain with another relevant domain for this case (like “secondary motor dual-tasking” of carrying a tray or “ambient demands” such as dim lighting) will provide greater demands for walking adaptability. Furthermore, patients' self-report of limitations when walking under varying task and environmental constraints may assist the clinical administration of selected and most relevant domains, as recently suggested by a study that preliminarily validated a self-report measure of environmental challenges to correlate with direct observations of mobility disability in individuals with stroke [130]. Ultimately, the availability of a validated comprehensive multidomain assessment for walking adaptability after stroke will assist in objectively quantifying the unique limitations in walking adaptability individualized to a person with stroke.

Development of a comprehensive and rigorous clinical assessment for walking adaptability can be facilitated by utilization of a recently proposed integrated approach to assessment scale development [131, 132]. Traditionally, functional outcome measures in rehabilitation have suffered from the lack of a theoretical or conceptual framework to guide their development [133, 134]. The result is the lack of comprehensive measurement by a single assessment tool despite the availability of several outcome measures adding to measurement. The approach proposed by Velozo and colleagues [131, 132] provides a structured method to guide development of a comprehensive assessment scale based on contemporary testing principles of item response theory (IRT). IRT is a methodological approach for the development, psychometric analysis, and scoring of assessment scales and is becoming the benchmark for developing and analyzing assessment scales [135, 136]. Specifically, Velozo et al. (2012) provide a roadmap of an 8-stage mixed-methods (using qualitative and quantitative methods) approach for the development of a contemporary IRT-based assessment scale [131]. The review of clinical assessments presented here could contribute towards some of the qualitative stages of the 8-stage mixed-methods approach and provide a foundation for developing a comprehensive and sound clinical assessment for walking adaptability. Therefore, we recommend utilizing the 8-stage mixed-methods approach to achieve development of a comprehensive clinical assessment for walking adaptability after stroke.

**Figure 4:**
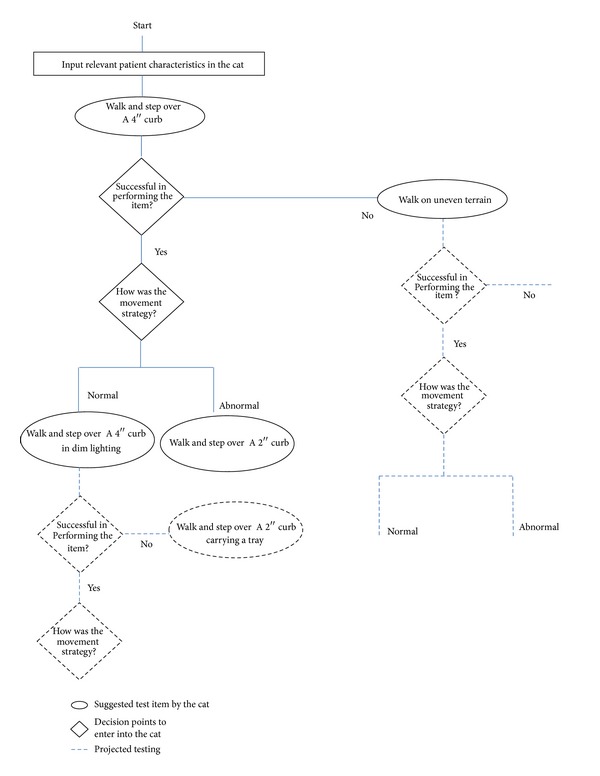
An illustrated example of an IRT-based computer adaptive test for walking adaptability after stroke. Development of an IRT-based assessment requires identification of a relevant pool of assessment items, conceptualizing a hierarchy of difficulty levels, and testing these assumptions using IRT methods. The IRT-based assessment may be administered using a computer adaptive format or an IRT-based static short form. This figure illustrates an example of hierarchical assessment items measuring walking adaptability in the terrain domain and administration of these items in an IRT-based computer adaptive test. Additionally, this example demonstrates incorporating assessment of not only “task performance”, but also the “quality of task performance” (i.e., movement strategy).

While a comprehensive and rigorous assessment tool of walking adaptability may be critical for targeted assessment, the familiar dilemma in outcome measurement development is the need to balance feasibility with comprehensiveness and precision. For instance, a comprehensive and rigorous assessment of walking adaptability may require several hierarchically developed items in each domain that can simulate the testing of real-life complexities of walking adaptability. However, a clinically feasible tool would necessitate efficient clinical administration of only relevant domains and those domain-specific items that match and further challenge the patient level. Contemporary methods of test administration such as IRT-based computer adaptive testing or IRT-based static short forms may offer a solution for developing a rigorous assessment of walking adaptability. For instance, an IRT-based computer adaptive test can provide efficient testing of only the most relevant items by providing item parameters to computer algorithms [131].  Figure 4 illustrates, as an example, items that could be included as part of an IRT-based computer adaptive test to assess walking adaptability specific to a patient's level of functioning. Recently developed IRT-based computer adaptive tests in rehabilitation can additionally serve as templates to guide the development of such a test [137–139]. Therefore, future work could consider application of similar approaches for individuals with stroke to develop a comprehensive yet patient-specific assessment of walking adaptability in this population.

We also recommend that a comprehensive multi-domain assessment of walking adaptability should include the assessment of movement strategies utilized to perform the adaptability tasks. The majority of the clinical assessments for walking function quantify the success of task performance, the amount of assistance required, and the time it takes to perform these tasks using ordinal rating scales (e.g., a 4-point scale like 0–4). While these are useful and validated approaches to quantify functional impairments, these methods provide limited information regarding* how* the task was accomplished. For instance, an individual after stroke may successfully clear an obstacle in a timely manner and yet do so, primarily utilizing compensatory approaches like heavily relying on an assistive device or using inefficient movement strategies such as excessive hip hiking and circumduction of the affected leg. Current walking assessments would fail to capture these compensatory movement strategies and in turn primarily grade the individual for simply the success in negotiating the obstacle. Assessment of how an individual performs a task provides insight into the extent to which a behavior is recovered and can directly guide effective recovery-based intervention strategies. Without this focus on the quality of task performance, clinical assessments are unable to distinguish between compensation and recovery and the extent to which rehabilitation approaches are impacting the reacquisition of normal motor behaviors [140]. Recently, there has been progress in this area and Behrman and colleagues developed a classification scale focused on task-specific recovery without compensations for individuals with a spinal cord injury [141]. Importantly, preliminary reports regarding use of this assessment (neuromuscular recovery scale) are very encouraging and have demonstrated its potential value in discriminating performance in the assessment of balance and walking. Similarly, a multidomain assessment of walking adaptability that measures the quality of task performance is critical to determine the effectiveness of therapeutic intervention in promoting recovery of walking adaptability and possibly motivate the design of novel recovery-based interventions for walking adaptability after stroke.

## 7. Conclusions

This review article examines the conceptual challenges for the measurement of walking adaptability and summarizes the current state of its clinical assessment. First, we have clarified the use of the terminology of walking adaptability based on the neural control model of walking to synchronize with the current recovery-based paradigms of walking rehabilitation. Second, we incorporated seven dimensions of community mobility proposed by Patla and Shumway-Cook and modified two dimensions to create overall nine unique domains of walking adaptability that can be applied to any ambulatory environment. Third, we have presented a thorough description of the existing clinical assessments of walking function that include items assessing walking adaptability and identify domains of adaptability captured by these assessments. Cumulatively, the evidence points to an urgent need for a comprehensive and rigorous approach for the clinical assessment of walking adaptability after stroke. We recommend that development of a comprehensive and rigorous clinical assessment may require (a) validation of hierarchically developed assessment items in each of the nine domains of walking adaptability, (b) utilization of contemporary measurement methods (such as IRT-based computer adaptive testing) for test administration and (c) assessment of “movement strategy” when performing adaptability tasks. Since accurate assessment is essential for the application and development of targeted interventions, future work in this area has great potential to contribute to breakthroughs in the rehabilitation of walking adaptability recovery after stroke and improved quality of life for individuals after stroke.

## Figures and Tables

**Table 1:** Domains of walking adaptability.

Domain∗	Definition∗
Obstacle negotiation^1^	Negotiating obstacles in the environment to prevent a collision between the lower limb and the obstacle, such as stepping over an obstacle
Temporal	Time constraints imposed on walking, such as needing to walk fast to cross a street or slow in a crowded mall
Cognitive dual-tasking^2^	Walking while attending to cognitive tasks, such as engaging in conversation while walking
Terrain demands	Walking on compliant or uneven surfaces that are not flat and firm, such as stairs, ramps, grass, and so forth
Ambient demands	Factors such as level of lighting, temperature, weather conditions, noise levels, and familiarity with surroundings
Postural transitions	Varying posture during walking, such as turning, bending down to pick an object while walking, and so forth
Motor dual-tasking^2^	Walking while attending to additional motor tasks, such as holding a glass of water while walking, picking up an object from the floor, and so forth
Physical Load	Carrying or interacting with a weighted object while walking, such as carrying a loaded back-pack, walking to open a heavy door, and so forth
Maneuvering in traffic^1^	Avoiding collision with static and dynamic objects by maneuvering the entire body, such as walking around other people, pets, vehicles, and so forth

*Modified from Patla and Shumway-Cook's conceptual framework defining dimensions of mobility [15].

^1^Originally categorized as “traffic density” in Patla and Shumway-Cook's dimensions of mobility.

^2^Originally categorized as “attentional demands” in Patla and Shumway-Cook's dimensions of mobility.

**Table 2:** Psychometric characteristics of the performance-based clinical assessments of walking function.

Assessment	Psychometric properties	Subjects	Results
DGI (Lin et al. 2010 [71])	Sensitivity, TrT, and C-validity	45 after stroke, 48 for TrT	MDC = 4 points, TrT ICC = 0.91–0.97, validity DGI wrt 4-item DGI and FGA *r* > 0.91

DGI (Jonsdottir and Cattaneo 2007 [72])	TrT, InterRR, and C-validity	25 postchronic stroke	TrT ICC = 0.96, InterRR ICC = 0.96, validity DGI wrt BBS *r* = 0.83, wrt ABC *r* = 0.68

DGI (Romero et al. 2011 [73])	Sensitivity	42 community-dwelling elderly	MDC = 2.9 points

DGI (Shumway-Cook et al. 1997 [74])	C-Validity	44 community-dwelling elderly	DGI wrt BBS *r* = 0.67, wrt balance self-perceptions test *r* = 0.76

DGI Danish version (Jønsson et al. 2011 [75])	InterRR, IntraRR	24 hospitalized adults 26 adults in outpatient rehab	Hospitalized InterRR ICC = 0.92, IntraRR ICC = 0.90; outpatients InterRR ICC = 0.82, IntraRR ICC = 0.89

DGI (Huang 2011)	Sensitivity, TrT	72 adults with PD	MDC = 2.9 points, TrT ICC = 0.84

DGI (Cakit et al. 2007 [76])	C-validity	31 adults with PD	DGI wrt UPDRS motor subscale *r* = −0.57, wrt history of falls *r* = 0.64

DGI (Hall and Herdman 2006 [77])	TrT	16 adults with vestibular disorders	TrT ICC = 0.86

DGI (Whitney et al. 2000 [78])	C-validity	30 adults with vestibular disorders	DGI wrt BBS *r* = 0.71

DGI (Marchetti et al. 2014 JNPT [79])	Sensitivity	326 adults with balance and vestibular disorders	MDC = 4 points

FGA (Lin et al. 2010 [71])	Sensitivity, TrT, Cand -validity	45 after stroke	MDC = 4.2 points, TrT ICC = 0.95, FGA wrt 10MWT *r* = −0.66, wrt PASS *r* = −0.83

FGA German version (Thieme et al. 2009 [80])	InterRR, IntraRR, and C-validity	28 after stroke	InterRR ICC = 0.94, IntraRR ICC = 0.97, FGA wrt FAC *r* = 0.83, wrt gait speed *r* = 0.82, wrt BBS *r* = 0.93, wrt RMI *r* = 0.85, wrt Barthel index *r* = 0.71

FGA (Walker et al. 2007 [81])	InterRR	200 healthy adults	InterRR ICC = 0.93

FGA (Leddy et al. 2011 [82])	TrT, InterRR	24 adults with PD, 15 for InterRR	TrT ICC = 0.91, InterRR ICC = 0.93

FGA (Wrisley and Kumar 2010 [83])	C-validity	35 community-dwelling elderly	FGA wrt BBS *r* = 0.84, wrt TUGT *r* = 0.84, wrt ABC scale *r* = 0.53

FGA (Ellis et al. 2011 [84])	C-validity	262 adults with PD	FGA wrt BBS *r* = 0.77, wrt PDQ-39 *r* = −0.57, wrt functional reach *r* = 0.52

FGA (Wrisley et al. 2004 [85])	InterRR, IntraRR, and C-validity	6 adults with vestibular disorders	InterRR ICC = 0.84, IntraRR ICC = 0.83, FGA wrt ABC scale *r* = 0.64, wrt TUGT *r* = −0.50, wrt DGI *r* = 0.80, wrt dizziness handicap inventory *r* = −0.64, wrt perception dizziness symptoms *r* = −0.70

FGA (Marchetti et al. 2014 JNPT [79])	Sensitivity	326 adults with balance and vestibular disorders	MDC = 6 points

mEFAP (Liaw et al. 2006 [86])	TrT, C-validity	20 postchronic stroke for TrT 40 postsubacute stroke for C-Validity	TrT ICC = 0.99, mEFAP wrt Barthel Index *r* = −0.52, wrt Rivermead Index *r* = −0.78

mEFAP (Baer and Wolf 2001 [87])	TrT, InterRR, and C-validity	26 after stroke	TrT ICC = 0.99, InterRR ICC = 0.99, mEFAP wrt BBS *r* = −0.70, wrt Functional assessment measure *r* = −0.78

SCI-FAP (Musselman and Yang 2014 [88])	Sensitivity	20 adults with incomplete SCI	MDC = 96 points

SCI-FAP (Musselman et al. 2011 [89])	TrT, InterRR	22 adults with incomplete SCI	TrT ICC = 0.98, InterRR ICC = 1.0,

Hi-MAT (Williams et al. 2006 [90])	Sensitivity, C-validity	103 adults with BI	MDC = +4 points, −2 points, Hi-MAT wrt motor FIM *r* = 0.53, wrt gross function rivermead motor assessment *r* = 0.87

Hi-MAT (Williams et al. in PTJ 2006 [91])	TrT, InterRR	103 adults with BI 20 adults with BI for TrT 17 adults with BI for InterRR	TrT ICC = 0.99, InterRR ICC = 0.99

CB&M (Balasubramanian 2014 [92])	InterRR, IntraRR, and C-validity	40 community dwelling elderly 37 for C-validity wrt 6MWT 36 for C-validity wrt gait speed	InterRR ICC = 0.95, IntraRR ICC = 0.96, CB&M wrt DGI *r* = 0.79, wrt BBS *r* = 0.87, wrt SPPB *r* = 0.75, wrt 6MWT *r* = 0.71, wrt TUGT *r* = −0.69, wrt gait speed *r* = 0.65, wrt ABC *r* = 0.47, wrt functional reach test *r* = 0.35

CB&M (Inness et al. 2011 [93])	C-validity	35 adults with BI	CB&M wrt gait velocity *r* > 0.67, wrt ABC *r* = 0.60

CB&M (Wright et al. 2010 [94])	Sensitivity, TrT, and InterRR	32 youths with BI	MDC = 13.5% points, TrT ICC = 0.90, InterRR ICC = 0.93

CB&M (Howe et al. 2006 [95])	TrT, InterRR, and IntraRR	32–36 adults with BI	TrT ICC = 0.98, InterRR ICC = 0.98, IntraRR ICC = 0.98

CB&M (Knorr et al. 2010 [96])	C-validity	44 after stroke	CB&M wrt Chedoke McMaster stroke assessment *r* = 0.63 (leg), *r* = 0.61 (foot)

WIT (Bandinelli et al. 2006 [97])	TrT	30 community-dwelling elderly	TrT ICC ≥ 0.75 for 13 of 14 items

SWOC (Rubenstein et al. 1997 [31])	TrT, InterRR, and C-validity	58 community-dwelling elderly men	TrT ICC = 0.93, InterRR ICC 0.81–1.0, obstacle course-R wrt gait velocity *r* = 0.61, wrt 6MWT *r* = 0.56, wrt POMA Gait score 0.52, wrt, POMA balance *r* = 0.42

Obstacle course (Means 1996 [32])	C-validity	237 community-dwelling elderly	Obstacle course-M wrt medical conditions *r* = −0.41, wrt number of medications *r* = −0.29

SOMAI (Tang et al. 1998 [98])	C-validity	27 community-dwelling elderly	SOMAI normal vision condition wrt 6 SOT conditions *r* = 0.21–0.53, SOMAI focal vision wrt 6 SOT conditions *r* = 0.20–0.59

MTT∗∗			

TrT, test-retest reliability; C-validity, criterion validity; InterRR, intertester reliability; IntraRR, intratester reliability DGI, dynamic gait index; FGA, functional gait assessment; mEFAP, modified Emory functional ambulation profile; SCI-FAP, spinal cord injury functional ambulation profile; Hi-MAT, high-level mobility assessment test; CB&M, community balance and mobility scale; SOMAI, sensory-oriented mobility assessment instrument; WIT, walking InCHIANTI toolkit; SWOC, standardized walking obstacle course developed by Rubenstein and colleagues; obstacle course, obstacle course developed by Means and colleagues; MTT, multiple task test; PD, Parkinson's disease; BI, brain injury; SCI, spinal cord injury; wrt, with respect to; MDC, minimal detectable change; 6MWT, 6-minute walk test; BBS, Berg balance scale; ABC, activities-specific balance confidence scale; UPDRS, unified Parkinson's disease rating scale; PASS, posture assessment scale for stroke patients; TUGT, timed up and go test; RMI, Rivermead mobility index; POMA, Tinetti performance-oriented mobility assessment; FIM, functional index measure; SPPB, short physical performance battery.

∗∗Developed for persons with Parkinson's disease. Quantitative data and strategies for persons with Parkinson's disease are reported. However, no specific psychometric characteristics have been reported.

**Table 3:** Domains of walking adaptability captured by performance-based clinical assessments of walking function.

Clinical assessment items		Domains of walking adaptability∗								
		ON	TM	CT	TR	AM	PT	MT	PL	TF
DGI										
2	Change speed		TM							
3	Horizontal head turns						PT			
4	Vertical head turns						PT			
5	Gait and pivot turn						PT			
6	Step over obstacle	ON								
7	Step around obstacle									TF
8	Stairs				TR					
FGA										
** 2**	**Change speed**		**TM**							
** 3**	**Horizontalhead turns**						**PT**			
** 4**	**Verticalhead turns**						**PT**			
** 5**	**Gait andpivot turn**						**PT**			
** 6**	**Step over obstacle**	**ON**								
7	Narrow BOS						PT			
9	Ambulate backwards						PT			
** 10**	**Stairs**				**TR**					
mEFAP										
2	Carpet				TR					
3	Up and go						PT			
4	Step over and around obstacles	ON								TF
** 5**	**Stairs**				**TR**					
SCI-FAP										
** 1**	**Carpet**				**TR**					
** 2**	**Up and go**						**PT**			
** 3**	**Obstacles**	**ON**								
** 4**	**Stairs**				**TR**					
5	Carry								PL	
6	Step Up	ON								
7	Door						PT	MT		
Hi-MAT										
2	Walk backwards		TM				PT			
3	Walk on toes		TM				PT			
4	Walk over obstacle	ON	TM							
** **10/12	**Stairs/dependant**				**TR**					
** **11/13	**Stairs/independent**				**TR**					
CB&M										
2	Tandem walk						PT			
6	Crouch and walk						PT	MT		
8	Walk and look						PT			
10	Forward to Backward walk		TM				PT			
11	Walk, look, and carry						PT	MT		
12	Descending stairs				TR					
SOMAI										
6/7	Cushion 1-NV				TR					
8/9	Cushion 2-NV				TR					
16/17	Cushion 1-FV				TR	AM				
18/19	Cushion 2-FV				TR	AM				
WIT										
2	4 m walk fast		TM							
3	4 m BOS 25 cm		TM				PT			
4	4 m BOS 15 cm		TM				PT			
5	7 m walk fast		TM							
6	7 m walk long steps						PT			
7	7 m fast walk obstacles	ON	TM							
8	7 m fast walk obstacles dim light	ON	TM			AM				
9	7 m usual walk carry package							MT		
10	7 m usual pace naming objects			CT						
11	7 m usual pace pick up 1/3 objects						PT	MT		
12	400 m fast walk		TM							
13	60 m fast walk weighted jacket		TM						PL	
SWOC										
** 1**	**Tandem walk **						**PT**			
2	Balance ladder with foam	ON			TR					
3	Ramp and stairs				TR					
4	Pick up empty box						PT	MT		
5	Miniblind						PT			
** 6**	**Step over a block**				**TR**					
OC										
1	Door opening						PT	MT		
2	Turf				TR					
3	Objects	ON								
** 4**	**Carpet**				**TR**					
** 5**	**Low steps**				**TR**					
6	Pine Bark				TR					
7	Cones									TF
8	Sand				TR					
9	Chair						PT			
** 10**	**Steep steps**				**TR**					
11	Upramp				TR					
12	Downramp				TR					
MTT										
1	Stand Up, walk, turn, and sit down						PT			
2	Item 1 + answer questions			CT			PT			
3	Item 1 + avoid obstacles	ON					PT			TF
4	Item 1 + carry an empty tray						PT	MT		
5	Item 1 + carry tray of 2 eggs, 1 rolling egg						PT	MT		
6	Item 1 + slippery shoes				TR		PT			
7	Item 1 + tip the floor halfway						PT			
8	Item 1 + wear sunglasses in dim light					AM	PT			

Items represented in bold are redundant across assessments.

Abbreviations: DGI, dynamic gait index; FGA, functional gait assessment; mEFAP, modified Emory functional ambulation profile; SCI-FAP, spinal cord injury functional ambulation profile; Hi-MAT, high-level mobility assessment test; CB&M, community balance and mobility scale; SOMAI, sensory-oriented mobility assessment instrument; WIT, walking InCHIANTI toolkit; SWOC, standardized walking obstacle course developed by Rubenstein and colleagues; obstacle course, obstacle course developed by Means and colleagues; MTT, multiple task test; ON, obstacle negotiation; TM, temporal demands; CT, cognitive dual-tasking; TR, terrain demands; AM, ambient demands; PT, postural transitions demands; MT, motor dual-tasking; PL, physical load; TF, maneuvering in traffic; BOS, base of support; NV, normal vision; FV, focal vision.

∗The domains for walking adaptability have been adapted from earlier work by Patla and Shumway-Cook [15].
